# Participation in Tourism-Oriented Nature-Based Physical Activity and Compliance With WHO Physical Activity Guidelines: A Cross-Sectional Analysis of the 2024 Sports Life Survey in Japan

**DOI:** 10.7759/cureus.87063

**Published:** 2025-06-30

**Authors:** Shinyu Kise

**Affiliations:** 1 Department of Tourism and Health Studies (Sponsored by Ryukyuseimeisaiseikai), Institute for Tourism and Health, Naha, JPN

**Keywords:** cross-sectional study, japan, nature-based physical activity, outdoor sports, perceived insufficient exercise, self-rated health, tourism, who physical activity guidelines

## Abstract

Background: Nature-based physical activity (NBPA), outdoor sports conducted in natural (green or blue) environments, can simultaneously promote public health and regional tourism.

Aim: This study examined whether participation in tourism-oriented NBPA is associated with (i) attainment of the 2020 World Health Organization (WHO) aerobic activity guidelines, (ii) perceived insufficient exercise, and (iii) self-rated health (SRH) in a nationally representative Japanese sample. We hypothesized that NBPA participants would show higher compliance with WHO physical activity guidelines, more favorable subjective health ratings, and lower perceived exercise insufficiency than non-participants, underscoring NBPA's potential as a public health strategy.

Methods: This secondary analysis used data from the Sports Life Survey 2024 (unweighted n = 2,896; weighted estimates reported). NBPA was defined as engaging at least once in the previous 12 months in one or more outdoor sports conducted in natural settings. Weighted logistic regression models were estimated in Python 3.11 (pandas 2.2.2, statsmodels 0.14.2, surveyweights 0.7.0; Python Software Foundation, Fredericksburg, VA, US) to compute adjusted odds ratios (Adj-ORs) for each outcome, controlling for age, sex, and body mass index (BMI).

Results: NBPA participation was reported by 68.1% of respondents. NBPA participants had significantly higher odds of meeting the WHO guideline (Adj-OR = 2.38, 95% CI 2.01-2.80, p < 0.001) but showed no significant differences in perceived insufficient exercise (Adj-OR = 0.97, 0.80-1.19) or SRH (Adj-OR = 1.13, 0.95-1.36).

Conclusions: Even occasional NBPA more than doubles the likelihood of meeting physical activity guidelines; however, infrequent participation does not appear to influence perceived exercise adequacy or overall health status. Public policies promoting regular, sustained NBPA engagement may yield broader health benefits and serve as models for integrating public health and tourism strategies globally.

## Introduction

Physical inactivity is a leading global risk factor for non-communicable diseases (NCDs) and premature mortality. An estimated 28% of adults worldwide fail to meet minimum activity recommendations, contributing substantially to the burden of ischemic heart disease, type 2 diabetes, breast and colon cancers, and stroke [1-3]. To address this, the World Health Organization (WHO) recommends that adults engage in 150-300 minutes per week of moderate-intensity aerobic activity, or 75-150 minutes per week of vigorous-intensity activity, or an equivalent combination [4]. The WHO’s Global Action Plan on Physical Activity (GAPPA) further aims for a 15% relative reduction in physical inactivity by 2030 [5]. However, recent national surveillance data indicate that only about one-third of Japanese men and one-quarter of Japanese women currently meet these guidelines [6], highlighting the urgent need for innovative, population-wide strategies to promote physical activity.

Nature-based physical activity (NBPA), defined as purposeful movement in outdoor green or blue environments such as forests, mountains, rivers, or coastal areas, has gained increasing attention as an accessible and enjoyable approach to increasing activity levels. Evidence suggests that NBPA offers additional physiological and psychological benefits beyond those of equivalent indoor or urban exercise [7-9]. Systematic reviews and meta-analyses show that even a single bout of “green exercise” can lead to greater acute improvements in mood and systolic blood pressure than indoor activities [10,11], while regular exposure to nature is prospectively associated with lower all-cause mortality and reduced depression risk [8,12].

Framing NBPA as a form of active tourism further enhances its value by aligning public health goals with regional economic development. Tourists engaging in outdoor recreation often spend more and stay longer than average visitors, creating economic incentives for local governments and businesses to promote NBPA [13,14]. Simultaneously, structured initiatives such as “park prescription” programs have shown that improving access to, and social support for, natural settings can increase moderate-to-vigorous physical activity among sedentary individuals [15]. Collectively, these findings suggest that tourism-oriented NBPA may offer a scalable, cross-sector strategy for boosting population physical activity and reducing the global NCD burden.

While international research supports the link between NBPA and greater physical activity levels, as well as improved well-being [8,9,16,17], large-scale epidemiological data from Asian populations remain limited. Leveraging data from the nationally representative Sports Life Survey 2024, the present study examines whether participation in tourism-oriented NBPA is associated with (i) attainment of the 2020 WHO aerobic activity guidelines, (ii) perceived insufficient exercise, and (iii) self-rated health (SRH) among Japanese adults. We hypothesized that NBPA participation would be positively associated with guideline compliance, better subjective health outcomes, and lower perceived exercise insufficiency. These findings carry important implications for public health promotion, tourism policy, and international models that seek to integrate nature-based tourism into strategies for improving population health.

## Materials and methods

Study design, setting, and dates

This cross-sectional secondary analysis utilized data from the Sports Life Survey 2024, an annual, nationally representative questionnaire administered by the Sasakawa Sports Foundation (SSF). Data collection was conducted from June 7 to July 7, 2024, across all 47 prefectures of Japan, using self-administered questionnaire survey by drop-off and pick-up method. Survey weights were created using post-stratification based on age, gender, and prefecture distributions from the 2020 National Census.

Ethical approval and data permission

The anonymized dataset is securely stored at Ryusei Hospital, and ethical approval was granted by the Ethics Committee of Ryusei Hospital, General Incorporated Foundation Ryukyu Seimeisaikai (Approval No. 202501; dated May 1, 2025). The official approval letter in both Japanese and English versions is provided.

Secondary use of the SSF data was authorized on April 30, 2025 (Agreement No. SL2024-A-002). The dataset is cited as: Sasakawa Sports Foundation. Sports Life Survey 2024 [dataset]. Tokyo: SSF; 2024.

Participants

From the original sample of 3,000 respondents, adults were eligible if they met the following inclusion criteria: (i) age ≥ 18 years and (ii) provided complete data for NBPA exposure, all three outcomes, age, sex, and body mass index (BMI). Exclusion criteria were implausible BMI values (< 12 or > 60 kg/m²) or any missing key variable, yielding a final analytic sample of 2,896 adults (unweighted).

Exposure variable

NBPA participation was defined as a “yes” response to having engaged in at least one of 13 outdoor sports typically conducted in natural settings (e.g., hiking, trail running, canoeing/kayaking, road cycling, open-water swimming, mountaineering, skiing, surfing, recreational fishing) during the past 12 months. NBPA participation was coded dichotomously (1 = participant; 0 = non-participant). A binary definition was adopted due to the lack of detailed data on frequency, duration, or intensity of activity.

Outcome measures

Outcomes measures were coded as binary variables: (1) WHO guideline attainment, meeting the 2020 WHO recommendation of ≥600 metabolic equivalent of task (MET)-min/week of moderate-to-vigorous physical activity; (2) perceived insufficient exercise, self-report that current exercise is “very” or “somewhat” insufficient; and (3) SRH, self-classification of overall health as “very healthy/healthy” versus “not very healthy/unhealthy” (binary).

Covariates

Age (years, continuous), sex (male/female), and BMI (kg/m², continuous) were included a priori as potential confounders.

Statistical analysis

Analyses were performed in Python 3.11, using pandas 2.2.2, statsmodels 0.14.2, and surveyweights 0.7.0 (Python Software Foundation, Fredericksburg, VA, US). Descriptive statistics were reported as weighted means ± SD or weighted percentages with unweighted n, and group comparisons were conducted using survey-weighted t-tests (for continuous variables) or Wald χ² tests (for categorical variables).

Multivariable Analysis

Three separate survey-weighted logistic regression models estimated the association between NBPA participation and each binary outcome (WHO guideline, perceived insufficient exercise, SRH). All models used replicate weights and HC3 robust standard errors and were adjusted for age, sex, and BMI.

Inference

Wald χ² statistics were used to compute two-sided p-values, with statistical significance defined as p < 0.05.

Sensitivity Analysis

Multiple imputation by chained equations (MICE) using miceforest (version 6.0.4) was applied for missing auxiliary covariates (none of the primary variables). Imputed and complete-case estimates were virtually identical (data not shown).

To support reproducibility, exact survey wording, detailed coding procedures, and analysis scripts will be provided in the supplementary materials upon publication.

## Results

Characteristics of the study population are summarized in Table 1.

**Table 1:** Weighted sample characteristics (unweighted counts in parentheses) All values are weighted estimates; counts (n) are unweighted. NBPA: nature-based physical activity, SRH: self-rated health.

Characteristic	Total (N = 2896)	NBPA (n = 1970)	Non-NBPA (n = 926)
Age, years ± SD	50.5 ± 17.1	48.9 ± 16.5	53.8 ± 17.9
Men, n (%)	1452 (50.1)	1030 (52.3)	422 (45.5)
Women, n (%)	1444 (49.9)	940 (47.7)	504 (54.5)
BMI, kg/m² ± SD	22.8 ± 3.6	22.7 ± 3.5	23.0 ± 3.7
Meets WHO guidelines, n (%)	1466 (50.6)	1132 (57.5)	334 (38.2)
Perceives insufficient exercise, n (%)	2290 (79.1)	1540 (78.3)	750 (79.7)
Good/very good SRH, n (%)	2086 (72.4)	1442 (73.2)	644 (71.7)

A total of 2,896 adults met the inclusion criteria (weighted to reflect the national population). Of these, 1,970 respondents (weighted 68.1%) reported engaging in at least one episode of tourism-oriented NBPA in the past 12 months.

NBPA participants were significantly younger (48.9 ± 16.5 years) than non-participants (53.8 ± 17.9 years; survey-weighted t = 9.4, p < 0.001) and more likely to be male (52.3% vs 45.5%; Wald χ² = 12.7, p < 0.001). Mean BMI differed minimally between groups (difference = 0.3 kg/m²) and was not statistically significant (p = 0.09). Compliance with the 2020 WHO aerobic activity guideline was markedly higher among NBPA participants (57.5%) compared to non-participants (38.2%; Wald χ² = 76.1, p < 0.001). In contrast, the prevalence of perceiving insufficient exercise was high in both groups (≈79%) and did not differ significantly (p = 0.58). The proportion of adults reporting good or very good SRH exceeded 70% in both groups, with no meaningful difference (p = 0.21).

Multivariable logistic regression outcomes are presented in Table 2.

**Table 2:** Adjusted odds ratios for the study outcomes Models incorporate post-stratification survey weights and HC3 robust standard errors. Adj-OR: adjusted odds ratio; SRH: self-rated health.

Outcome	Adj-OR (95% CI)	Wald χ² p-value
WHO guideline attainment	2.38 (2.01-2.80)	<0.001
Perceived insufficient exercise	0.97 (0.80-1.19)	0.78
Good/very good SRH	1.13 (0.95-1.36)	0.17

After adjustment for age, sex, and BMI, NBPA participation was independently associated with significantly higher odds of meeting the WHO physical activity guidelines (Adj-OR = 2.38, 95% CI 2.01-2.80; Wald χ² = 72.9, p < 0.001). However, NBPA was not significantly associated with perceived insufficient exercise (Adj-OR = 0.97, 95% CI 0.80-1.19, p = 0.78) or with good/very good SRH (Adj-OR = 1.13, 95% CI 0.95-1.36, p = 0.17). Sensitivity analyses using multiple imputations for missing covariates yielded nearly identical results (data not shown).

In summary, tourism-oriented NBPA is highly prevalent among Japanese adults and is strongly associated with objective compliance with WHO physical activity recommendations. However, occasional engagement alone does not appear sufficient to influence subjective perceptions of exercise adequacy or their overall SRH.

## Discussion

Principal findings

In this nationally representative sample, participation in tourism-oriented NBPA was common (68%) and was independently associated with more than twice the odds of meeting the 2020 WHO aerobic activity guidelines. By contrast, NBPA was not associated with perceived exercise insufficiency or SRH. These findings suggest that even infrequent outdoor recreation may substantially contribute to meeting objective activity benchmarks, but may lack the frequency or duration needed to influence subjective perceptions of health or exercise adequacy.

Comparison with previous literature

Our finding that NBPA promotes compliance with physical activity guidelines aligns with meta-analyses reporting higher total physical activity levels among individuals who engage in exercise in natural environments compared to those active exclusively indoors [8,16]. Prospective studies from Europe have shown that access to attractive green or blue spaces increases the likelihood of meeting recommended activity levels [17]. The strength of association observed in the present study (Adj-OR ≈ 2.4) is comparable to effect sizes observed in park-prescription trials providing structured outdoor exposure to sedentary adults [15].

In contrast, the lack of a significant association between NBPA and perceived exercise sufficiency or SRH mirrors findings from experimental studies where single or infrequent bouts of green exercise improved acute mood and cardiorespiratory fitness, but did not meaningfully affect broader well-being metrics [10,11]. A large English cohort study identified approximately 120 minutes of nature exposure per week as a threshold for improved SRH [17]; many respondents in our sample likely fell short of this threshold, as NBPA participation was defined by as little as one outing per year. Recent reviews suggest that sustained and regular contact with nature is essential for long-term mental health benefits, rather than sporadic experiences [18,19]. Additionally, urban ecological research highlights that regular access to nearby nature may be as influential as destination-based recreation in promoting outdoor activity frequency [20].

Our low participation threshold (≥1 outing per year) likely underestimates the exposure required to observe meaningful subjective health improvements. Emerging evidence indicates that weekly engagement may be necessary to yield substantial gains in perceived health and mental well-being. As such, promoting more frequent NBPA through structured initiatives may amplify its public health benefits.

Public health and tourism implications

NBPA sits at the intersection of health promotion and regional economic development. Outdoor-sport tourists typically exhibit higher daily expenditures and longer stays than average visitors, generating economic incentives for municipalities to expand recreational infrastructure [13,14]. Our results provide quantitative support for such investments: even sporadic engagement in outdoor sports nudges a sizable segment of the population toward guideline adherence. Local governments could scale up guided hikes, community trail-running events, or discounted equipment-rental schemes to convert episodic participation into weekly or monthly habits. Health sectors might collaborate by prescribing park visits, offering green exercise classes, integrating wearable device prompts, or encouraging at least weekly nature contact. Achieving ≥120 minutes per week of outdoor activity may represent a pragmatic behavioral target balancing feasibility with tangible subjective health gains.

Furthermore, our findings may serve as a valuable model internationally, illustrating how countries can strategically integrate NBPA into public health initiatives and tourism policies. Adopting similar cross-sector approaches could globally contribute to reducing physical inactivity and related NCDs.

Strengths and limitations

The strengths of this study include (i) a large, nationally representative sample with post-stratification weighting, (ii) alignment of outcome definitions with the 2020 WHO guidelines, and (iii) a transparent, reproducible Python workflow. Limitations merit consideration. First, the cross-sectional design precludes causal inference; active individuals may self-select into NBPA. Second, all exposure and outcome data were self-reported, potentially introducing recall and social desirability biases; however, validated WHO-aligned measurement scales help mitigate these biases. Third, the binary NBPA measure lacked detailed information on frequency, duration, and intensity of participation, limiting our ability to precisely assess dose-response relationships. Finally, despite adjustments for age, sex, and BMI, residual confounding by socioeconomic factors, educational attainment, job profile, nationality, ethnicity, or proximity to natural amenities may remain.

Future research

Future longitudinal studies and randomized controlled trials should specifically investigate the dose-response relationships between NBPA frequency, duration, intensity, and a broader range of health outcomes. Including diverse demographic factors such as socioeconomic status, education level, nationality, ethnicity, and detailed job profiles would enhance understanding of NBPA’s differential impacts and help design targeted interventions. Additionally, qualitative research exploring barriers and facilitators to regular NBPA participation, particularly among older adults and urban residents, would further inform tailored interventions. Finally, economic evaluations examining the cost-benefit ratios of tourism-driven outdoor recreation infrastructure would aid policymakers in balancing public health and fiscal priorities.

## Conclusions

Tourism-oriented NBPA is already practiced by more than two-thirds of Japanese adults and is independently associated with more than double the odds of meeting the 2020 WHO aerobic activity guidelines. This sizable behavioral advantage emerges even when NBPA is undertaken only occasionally (≥1 outing in the previous 12 months), underscoring the public health potential of Japan’s abundant natural and coastal resources. Yet, the same data reveal that infrequent NBPA alone does not appreciably alter individuals’ perceptions of exercise adequacy or improve overall SRH. Converging evidence suggests that a threshold of roughly 120 minutes of weekly outdoor activity is necessary to translate objective gains in guideline compliance into subjective health benefits. Therefore, transforming episodic outdoor recreation into regular, ideally weekly, engagement should be prioritized in future policy and program initiatives.

Our findings highlight NBPA as an effective intersection between public health promotion and sustainable tourism, presenting a practical, replicable framework for other nations seeking integrated strategies to reduce physical inactivity and associated NCDs globally.
